# Weight‐bearing 3D morphology of valgus knees: A comparative study within a varus dominant cohort

**DOI:** 10.1002/jeo2.70760

**Published:** 2026-05-15

**Authors:** Fangzhou Chi, Tomoharu Mochizuki, Keisuke Maeda, Hiroyuki Kawashima

**Affiliations:** ^1^ Division of Orthopedic Surgery Graduate School of Medical and Dental Sciences Niigata Japan; ^2^ Department of Orthopedic Surgery The First Affiliated Hospital of Harbin Medical University Harbin China

**Keywords:** knee osteoarthritis, lower‐limb alignment, morphology, valgus knee, varus knee

## Abstract

**Purpose:**

Using a weight‐bearing 3D assessment method to characterize the skeletal morphology and lower‐limb alignment of valgus knees, using a larger cohort of varus knees as a reference.

**Methods:**

We retrospectively reviewed imaging data of Knee osteoarthritis (KOA) patients (2010–2025), including 60 valgus and 264 varus knees classified by the coronal hip–knee–ankle (HKA). CT‐based 3D bone models were registered to biplanar weight‐bearing radiographs to quantify femoral and tibial morphological parameters and lower‐limb alignment under weight‐bearing conditions.

**Results:**

Compared with varus knees, valgus knees had greater femoral neck anteversion and larger condylar twist angle (both *p* < 0.05). The location of maximum tibial bowing was more proximal in varus knees than in valgus knees (*p* = 0.039). Distal tibial articular surface inclination showed opposite directions (*p* = 0.027). Coronal alignment revealed higher medial proximal tibial angle (MPTA) and lower lateral distal femoral angle (LDFA) in valgus knees and opposite findings in varus knees, resulting in significantly different arithmetic hip–knee–ankle (aHKA) phenotypes between the two groups (all *p* < 0.001). Axial alignment differed significantly, with relative tibial internal rotation in varus knees and external rotation in valgus knees (*p* < 0.001). Sagittal knee extension did not differ between groups.

**Conclusion:**

This study uses weight‐bearing 3D assessment technology to investigate valgus knees within a dataset dominated by varus knees, comparing differences in skeletal morphology and lower‐limb alignment between varus and valgus knees. These results indicate that, compared with varus knees, valgus knees exhibit demonstrate skeletal morphology and lower‐limb alignment across multiple planes. These findings provide new evidence supporting multiplanar differences in the phenotypic characterization of knee osteoarthritis.

**Level of Evidence:**

Level III.

**Abbreviations**
2D: two‐dimensional
3D: three‐dimensional
ACC: anatomical center curve
aHKA: arithmetic HKA
CTA: condylar twist angle
ER: external rotation
FTA: femorotibial angle
HKA: hip‐knee‐ankle
IR: internal rotation
KOA: knee osteoarthritis
LDFA: lateral distal femoral angle
MPTA: medial proximal tibial angle
PCA: posterior condylar angle
PCL: posterior condylar line
TEA: transepicondylar axis
TKA: total knee arthroplasty

## INTRODUCTION

Varus and valgus malalignment are common in knee osteoarthritis (KOA) and are associated with accelerated degeneration of the corresponding compartments [4]. Although varus deformity has been extensively investigated, valgus represents a distinct clinical and anatomical type in total knee arthroplasty (TKA). Despite numerous investigations into surgical techniques and soft tissue balancing strategies for valgus TKA, comprehensive three‐dimensional (3D) assessments of bone morphology and lower limb alignment under weight‐bearing conditions remain limited [1, 15, 23]. Importantly, inadequate correction in severe valgus deformity has been linked to a higher risk of prosthesis failure [14]. Therefore, a comprehensive understanding of the bone morphology and alignment characteristics of valgus knees is clinically important for developing personalized osteotomy strategies and optimizing postoperative lower‐limb alignment reconstruction [15].

Most clinical evaluations still rely on two‐dimensional (2D) long‐leg radiographs, which reliably quantify coronal alignment but provide limited information on sagittal or axial morphology and are susceptible to rotational positioning errors [4, 17, 19, 22]. CT‐based studies can describe 3D morphology, yet scans are typically obtained in a non–weight‐bearing posture and may not reflect functional alignment during standing [9, 11, 13]. Therefore, assessment methods that account for both weight‐bearing conditions and 3D perspectives are essential for accurately evaluating lower‐limb varus and valgus deformities.

To address this research gap, we developed an innovative weight‐bearing 3D lower‐limb alignment assessment system (Knee CAS; Lexi Co.), which integrates CT‐reconstructed 3D skeletal models with biplanar standing radiographs [3, 10, 16, 24]. Using 3D‐to‐2D registration technology, this system enables highly accurate automatic measurement of femoral and tibial 3D morphology and lower‐limb alignment under functional weight‐bearing conditions [10]. Using this method, our study aimed to compare the 3D morphology of the femur and tibia, together with lower‐limb alignment, between varus and valgus knees under weight‐bearing conditions. We tested two hypotheses: (1) valgus and varus knees would demonstrate distinct femoral and tibial morphological features across multiple planes and (2) 3D lower‐limb alignment under weight‐bearing conditions would differ between these two deformity patterns.

## MATERIALS AND METHODS

This retrospective study was approved by the Institutional Review Board of Niigata University (approval No.2020‐0448). All patients provided informed consent for the use of their imaging data. This research collected lower‐limb imaging data from patients with KOA who were treated at our institution between 2010 and 2025. The mechanical axes of the femur and tibia were defined in 3D space within the weight‐bearing coordinate system. The femoral mechanical axis connected the center of the femoral head to the knee center, and the tibial mechanical axis connected the knee center to the center of the talar dome. The HKA angle was calculated as the angle between these two mechanical axes. For classification purposes, the coronal component of this HKA was used to define varus and valgus alignment. After excluding cases with incomplete imaging, knees were categorized according to the coronal HKA angle: knees with HKA ≥ 180° (range: 180.21–208.78°) were classified as varus, and those with HKA < 180° (range: 146.09–179.51°) as valgus. Ultimately, 264 varus knees and 60 valgus knees were included.

**Figure 1:**
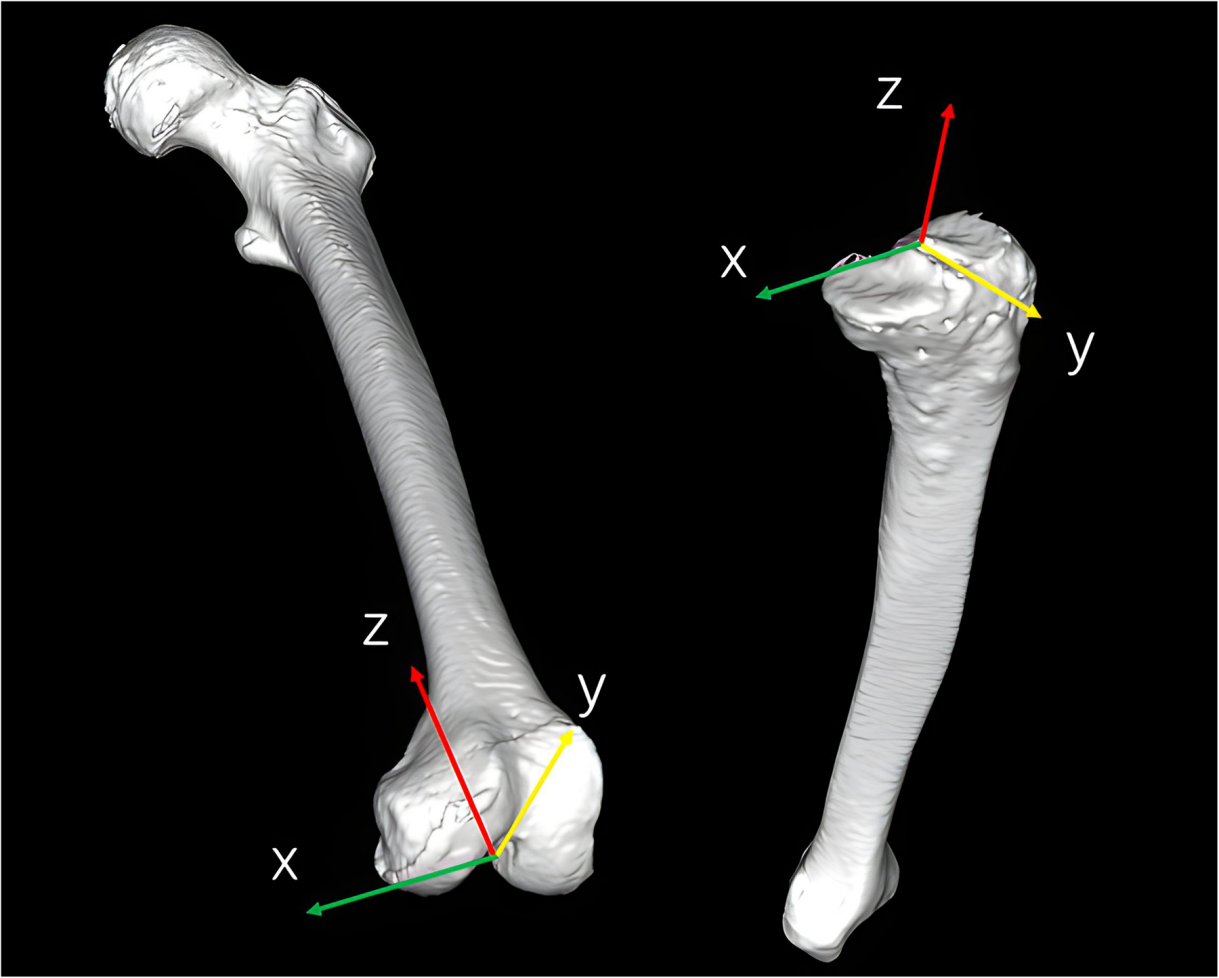
Schematic illustration of femoral and tibial coordinate system.

In the 3D alignment assessment system, digital femoral and tibial models were reconstructed from CT data (SOMATOM Sensation 16; Siemens Inc.) using visualization software (ZedView; Lexi Inc.), and anatomical coordinate systems were established according to validated definitions [16]. For the femoral coordinate system, the geometric center axis—connecting the centers of spherical models fitted to the medial and lateral posterior condyles—was defined as the *x*‐axis (positive to the right). The origin was defined as the midpoint between the centers of the posterior condylar spheres. The femoral *y*‐axis (positive anteriorly) was defined as the line perpendicular to the plane formed by the femoral head center and the two condylar sphere centers. The *z*‐axis (positive superiorly) was defined as the cross‐product of the *x*‐ and *y*‐axes. For the tibia, the *z*‐axis (positive superiorly) was defined as the line connecting the midpoint of the intercondylar eminence to the centers of the medial and lateral talar dome. The tibial *y*‐axis (positive anteriorly) was defined as the line perpendicular to the *z*‐axis passing through the medial aspect of the posterior cruciate ligament attachment. The tibial *x*‐axis (positive laterally) was defined as the cross‐product of the *z*‐ and *y*‐axes (Figure 1).

**Figure 2:**
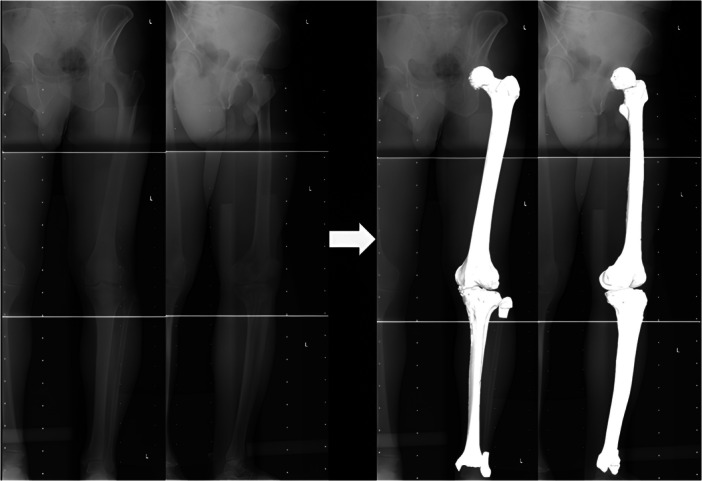
Biplanar computed radiography (CR) images of the entire lower extremities were taken in the weight bearing by standing position. The 3D digital bone models were projected onto the biplanar CR images by 3D‐to‐2D image registration technique.

Biplanar weight‐bearing long‐leg radiographs were acquired in a standardized standing position. 3D to 2D Image registration technology (KneeCAS; Lexi Inc.) [3, 10, 16, 24] was used to project the 3D digital bone models from CT data onto 2D radiographic images, incorporating anatomical coordinate systems along with morphological and alignment information. After registration, the system automatically calculated the relative positions of the femur and tibia and extracted 3D morphological and alignment parameters (Figure 2). The accuracy of the system was established as follows: three spherical markers were attached to each sawbone of the femur and tibia to determine the local coordinate system. Outlines of the 3D bone models were projected on extracted contours of each of the femur and tibia in frontal and oblique radiographic images. The 3D position of each model is restored by minimizing the difference between the projected contour line and the actual contour line. The median and maximum absolute errors in the relative position of the femur and tibia were 0.5 mm and 0.61°, and 1.6 mm and 1.5°, respectively. Inter‐observer and intra‐observer reproducibility errors were 1.9° and 0.8% [3, 10].

The evaluated parameters were as follows:

**Femoral morphology:** femoral bowing, anterior bowing, lateral bowing, location of maximum bowing, femoral neck anteversion, posterior condylar angle (PCA) and condylar twist angle (CTA).
**Tibial morphology:** tibial bowing, anterior bowing, lateral bowing, location of maximum bowing, tibial torsion and distal tibial articular surface inclination.
**Lower‐limb alignment:** femorotibial angle (FTA), MPTA, LDFA and aHKA. knee flexion/extension angle, and knee axial rotation angle.

**Figure 3:**
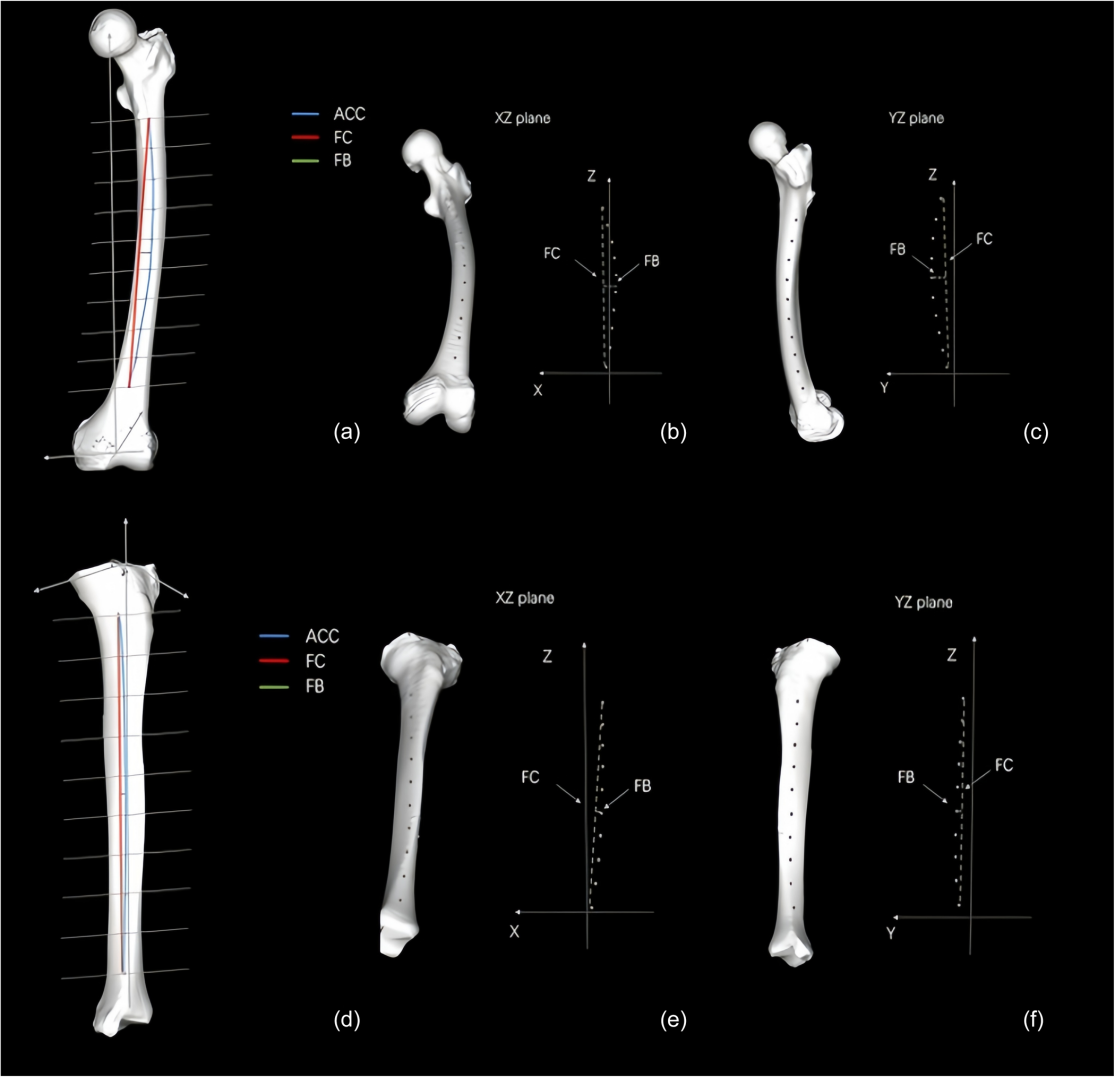
Measurement of femoral and tibial bowing in three dimensions. (a) Femoral bowing in 3D space was quantified by first defining the anatomical center curve (ACC) of the femur, obtained by connecting the center points of each cross‐section along the femoral axis. The straight line connecting the two endpoints of the ACC was defined as the FC. FB was measured as the maximum perpendicular distance from the ACC to the FC, and the bowing index was calculated as FB/FC × 100 (%). (b) Anterior femoral bowing was defined as the bowing of the ACC projected onto the femoral YZ plane. (c) Lateral femoral bowing was defined as the bowing of the ACC projected onto the femoral XZ plane. (d–f) Tibial bowing, anterior bowing, and lateral bowing were defined and calculated using the same method as for the femur.

**Figure 4:**
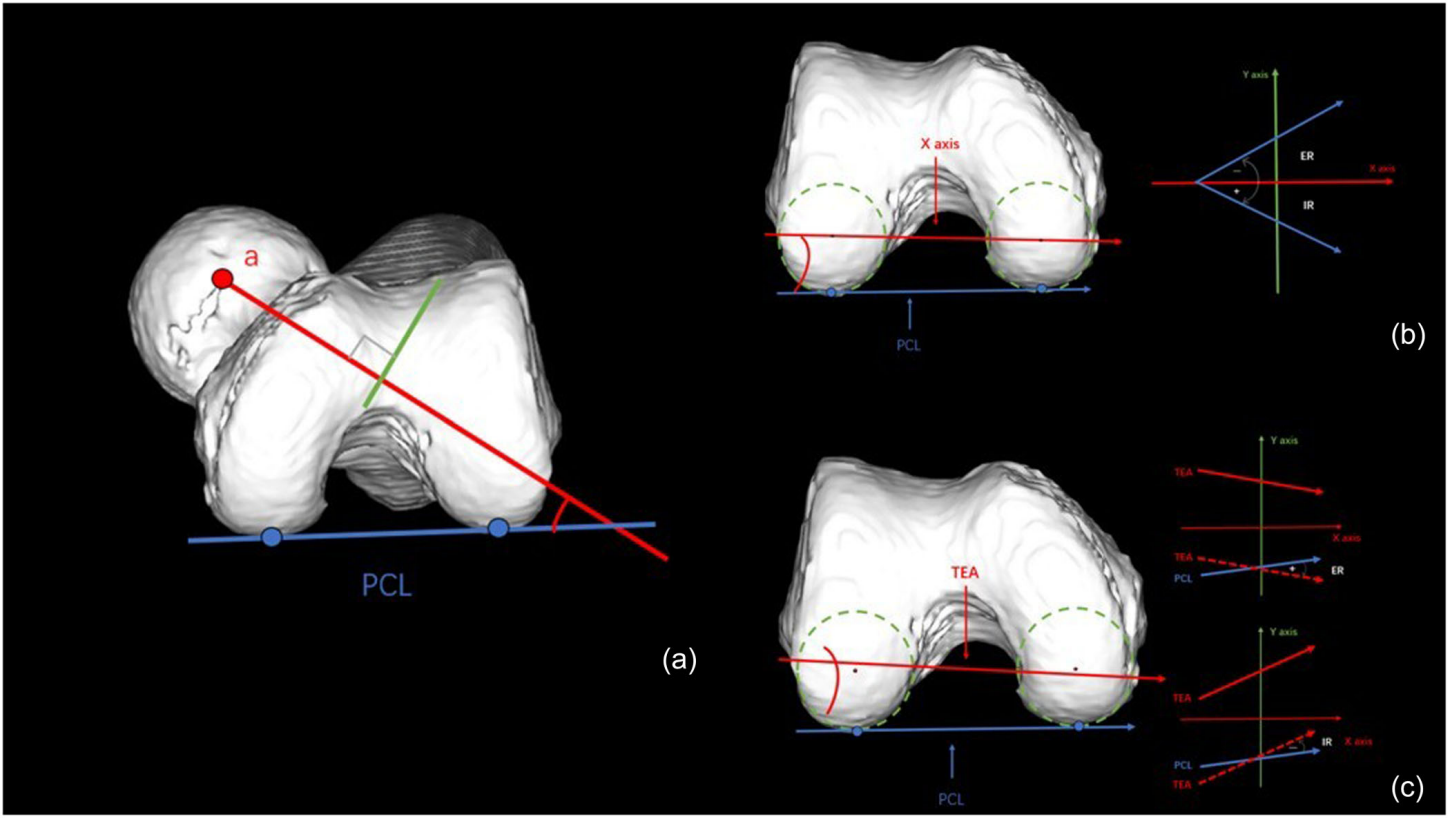
Measurement of femoral parameters. (a) Femoral neck anteversion was defined as the angle between a line (a) drawn perpendicular to the femoral shaft axis and the posterior condylar line (PCL), both projected onto the femoral XY plane. (b) The posterior condylar angle (PCA) was defined as the angle between the PCL and the femoral x‐axis in the XY plane, recorded as negative for internal rotation and positive for external rotation. (c) The condylar twist angle (CTA) was defined as the angle between the transepicondylar axis (TEA) and the PCL projected in the femoral XY plane.

To define femoral bowing in 3D space (Figure 3a), the anatomical center curve (ACC) was defined by connecting the center points of each cross‐section along the femoral axis; the straight line connecting the two endpoints of the ACC was defined as FC; and the maximum perpendicular distance from the ACC to the FC was measured as FB. Femoral bowing was calculated as FB/FC × 100 (%). Anterior bowing (Figure 3b) was defined as the bowing of the ACC projected onto the femoral YZ plane. Lateral bowing (Figure 3c) was defined as the bowing projected onto the XZ plane. Femoral neck anteversion (Figure 4a) was defined as the angle between a line (a) perpendicular to the femoral shaft axis and the posterior condylar line (PCL) projected in the XY plane. The PCA (Figure 4b) was defined as the angle between the PCL and the femoral *x*‐axis in the XY plane (negative for internal rotation, positive for external rotation). The CTA (Figure 4c) was defined as the angle between the transepicondylar axis (TEA) and the PCL in the femoral XY plane.

**Figure 5:**
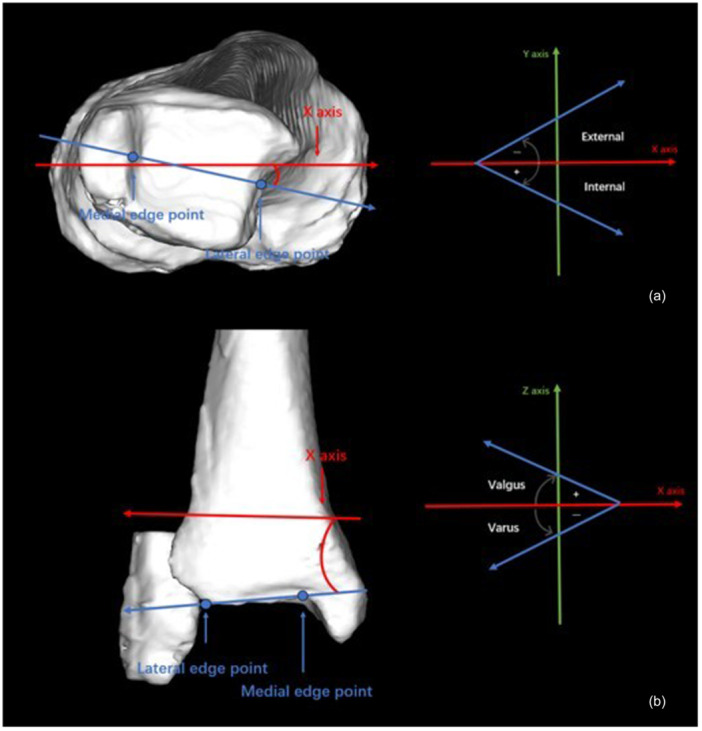
Measurement of tibial parameters. (a) Tibial torsion was defined as the angle between the distal articular edge line and the tibial *x*‐axis in the tibial XY plane, recorded as negative for internal torsion and positive for external torsion. (b) Distal tibial articular surface inclination was defined as the angle between the distal articular surface edge line and the tibial *x*‐axis in the XZ plane, recorded as negative for varus inclination and positive for valgus inclination.

Tibial bowing, anterior bowing, and lateral bowing were calculated using the same method as for the femur (Figure 3d–f). Tibial torsion (Figure 5a) was defined as the angle between the distal articular edge line and the tibial *x*‐axis in the XY plane (negative for internal torsion, positive for external torsion). Distal tibial articular surface inclination was defined as the angle between the distal articular surface edge line and the tibial *x*‐axis in the XZ plane (negative for varus, positive for valgus) (Figure 5b).

**Figure 6:**
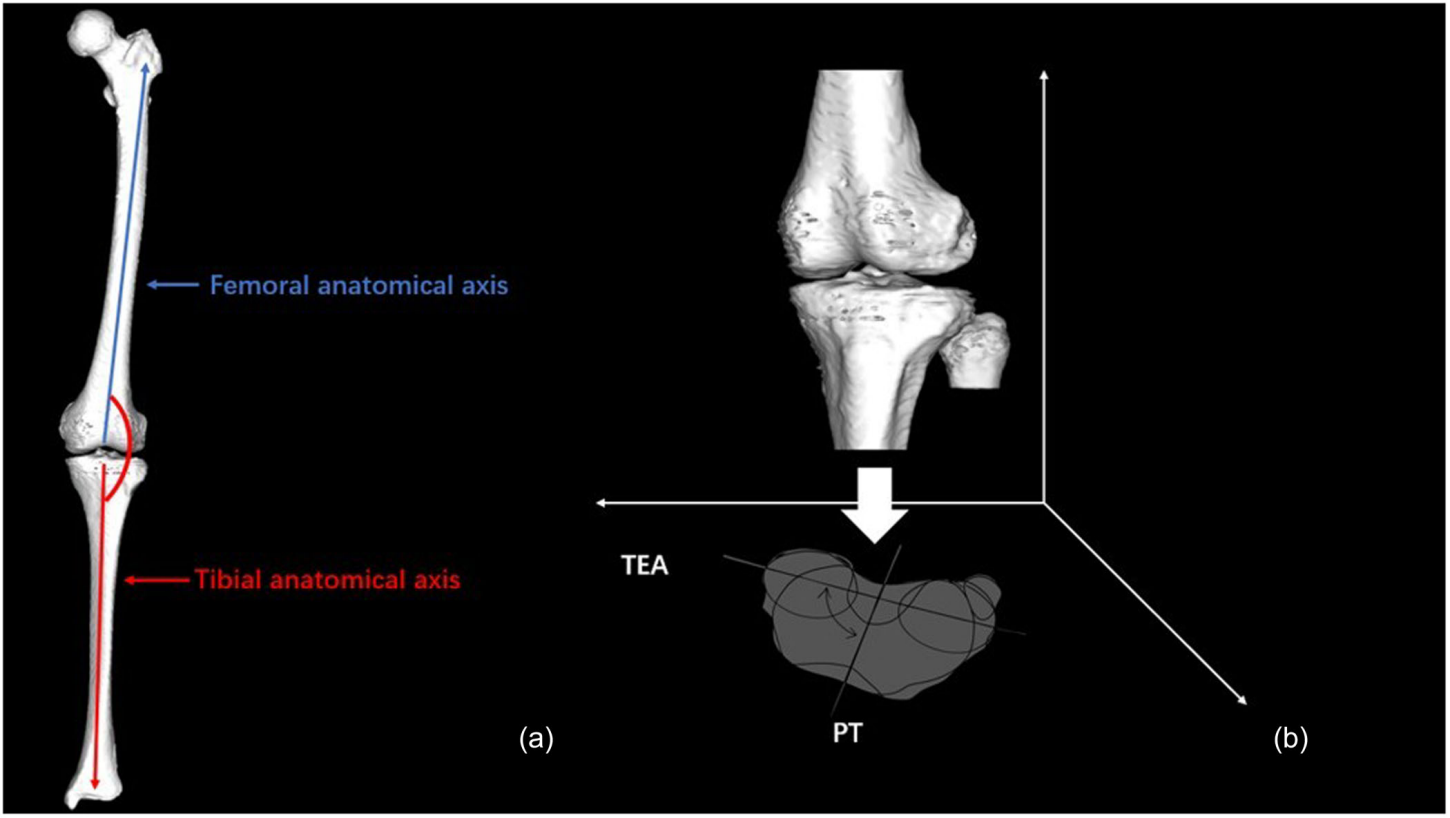
Measurement of alignment parameters. (a) The femorotibial angle (FTA) was defined as the angle between the anatomical axes of the femur and tibia in the femoral XZ plane. (b) The knee axial rotation angle was defined as the angle between the femoral transepicondylar axis (TEA) and PT axis (Akagi line) projected onto the tibial axial plane, where larger values indicate greater internal rotation of the tibia relative to the femur.

For alignment parameters, the FTA (Figure 6a) was defined as the angle between the femoral and tibial anatomical axes in the femoral XZ plane. The MPTA was defined as the medial angle between the tibial mechanical axis and the tibial plateau joint line in the coronal plane. The LDFA was defined as the lateral angle between the femoral mechanical axis and the distal femoral joint line in the coronal plane. The aHKA was calculated as aHKA = MPTA − LDFA, and negative values indicate varus, while positive values indicate valgus [8]. Knee axial rotation angle (Figure 6b) was defined as the angle between the femoral TEA and tibial PT axis (Akagi line) projected onto the tibial axial plane, where larger values indicate greater tibial internal rotation relative to the femur.

### Statistical analysis

Normality and homogeneity of variances were tested for each parameter within both groups. When the data were normally distributed with equal variances, intergroup differences were analyzed using the Student's *t*‐test; when the data were normally distributed but variances were unequal, the Welch's *t*‐test was applied. For data that did not follow a normal distribution, the Mann–Whitney *U* test was used. A *p* < 0.05 was considered statistically significant. For effect size estimation, Hedges'g was calculated for continuous variables that were normally distributed with equal variances, and Cliff's *δ* was used for nonnormally distributed variables. Post hoc power analyses were also conducted to evaluate the adequacy of the sample size. All statistical analyses were performed using SPSS version 27 (SPSS, Inc.).

## RESULTS

A total of 324 knees (264 varus, 60 valgus) from patients with KOA were analyzed. Detailed data are shown in Tables 1 and 2.

### Femoral morphology

No significant intergroup differences were observed in overall femoral bowing, anterior bowing, lateral bowing, and location of maximum bowing (Table 2). Femoral neck anteversion was significantly greater in valgus knees (36.0° ± 19.3°) than in varus knees (29.1° ± 10.1°, *p* = 0.004) (Tables 1 and 2). For distal femoral morphology, the CTA was also significantly larger in valgus knees (4.9° ± 3.7°) compared with varus knees (3.9° ± 2.8°, *p* = 0.016), indicating increased distal femoral external rotation in valgus knees, whereas the PCA did not differ between groups (Tables 1 and 2).

**Table 1:** Each data of evaluation parameters.

	Varus Group		Valgus Group	
Parameter	Mean	95% CI	Mean	95% CI
Femoral morphology				
Femoral bowing (%)	3.71	[3.58, 3.83]	3.65	[3.35, 3.94]
Femoral anterior bowing (%)	3.50	[3.40, 3.60]	3.34	[3.04, 3.65]
Femoral lateral bowing (%)	1.17	[1.01, 1.33]	1.26	[1.00, 1.52]
Location of maximal femoral bowing (%)	55.77	[54.85, 56.70]	53.79	[51.45, 56.13]
Femoral neck anteversion (°)	29.08	[27.86, 30.30]	36.40	[31.42, 41.38]
PCA (°; IR−, ER +)	−0.92	[−1.12, −0.72]	−1.22	[−1.68, −0.76]
CTA (°; IR−, ER +)	3.85	[3.51, 4.19]	4.86	[3.99, 5.72]
Tibial Morphology				
Tibial bowing (%)	2.94	[2.58, 3.30]	3.15	[2.41, 3.89]
Tibial anterior bowing (%)	1.99	[1.66, 2.32]	1.97	[1.43, 2.50]
Tibial lateral bowing (%)	2.02	[1.67, 2.36]	2.27	[1.58, 2.97]
Location of maximal tibial bowing (%)	50.80	[47.55, 54.05]	58.17	[51.75, 64.58]
Tibial torsion (°; internal −, external +)	12.70	[10.77, 14.63]	12.74	[8.10, 17.39]
Distal tibial articular surface inclination (°; varus −, valgus +)	0.91	[0.18, 1.65]	‐0.66	[‐2.05, 0.74]
Alignment parameter				
FTA (°)	186.04	[185.13, 186.96]	168.01	[165.57, 170.45]
MPTA (°)	81.90	[81.48, 82.31]	90.61	[88.55, 92.67]
LDFA (°)	89.56	[89.26, 89.86]	82.69	[81.24, 84.15]
aHKA (°)	−7.66	[−8.15, −7.17]	7.92	[5.66, 10.17]
Knee flexion/extension angle (°; flexion +, extension −)	16.48	[15.27, 17.69]	13.76	[9.88, 17.64]
Knee axial rotation angle (°)	91.84	[90.36, 93.33]	82.45	[78.37, 86.53]

*Note*: Data are reported as mean [95% CI].

Abbreviations: aHKA, arithmetic hip–knee–ankle angle (calculated as MPTA−LDFA); CI, confidence interval; CTA, condylar twist angle; ER, external rotation; FTA, femorotibial angle (>180° indicates varus, < 180° valgus); IR, internal rotation; LDFA, lateral distal femoral angle; MPTA, medial proximal tibial angle; PCA, posterior condylar angle.

**Table 2:** Comparison of alignment parameters between varus and valgus group.

Parameter	*p* value	Effect size	Power
Femoral morphology			
Femoral bowing	0.346	0.08	0.16
Femoral anterior bowing	0.086	0.14	0.42
Femoral lateral bowing	0.348	0.08	0.16
Location of maximal femoral bowing	0.119	0.13	0.36
Femoral neck anteversion	0.004*	0.24	0.85
PCA	0.132	0.12	0.34
CTA	0.016*	0.34	0.67
Tibial morphology			
Tibial bowing	0.982	0.00	0.05
Tibial anterior bowing	0.485	0.06	0.11
Tibial lateral bowing	0.606	0.04	0.08
Location of maximal tibial bowing	0.039*	0.17	0.57
Tibial torsion	0.729	0.03	0.06
Distal tibial articular surface inclination	0.027*	0.18	0.62
Alignment parameter			
FTA	<0.001*	0.95	1.00
MPTA	<0.001*	0.85	1
LDFA	<0.001*	0.8	1
aHKA	<0.001*	0.98	1
Knee flexion/extension angle	0.196	0.11	0.26
Knee axial rotation angle	<0.001*	0.37	1.00

*Note*: *p* < 0.05 was considered statistically significant.

Abbreviations: aHKA, arithmetic hip–knee–ankle angle (calculated as MPTA−LDFA); CTA, condylar twist angle; FTA, femorotibial angle (>180° indicates varus, <180° valgus); LDFA, lateral distal femoral angle; MPTA, medial proximal tibial angle; PCA, posterior condylar angle.

* indicates a statistically significant difference. Report the effect‐size index used (e.g., Cohen's *d*) and the *α* level assumed for statistical power.

### Tibial morphology

Overall, tibial bowing, anterior bowing, and lateral bowing were similar between groups. The location of maximum posterior tibial bowing was significantly more proximal in varus knees (50.8% ± 26.9%) than in valgus knees (58.2% ± 24.8%, *p* = 0.039; Hedges'*g* = 0.17) (Tables 1 and 2). There were no significant differences between groups in tibial torsion. However, distal tibial articular surface inclination showed opposite directions: lateral inclination in varus knees (0.9° ± 6.1°) and medial inclination in valgus knees (−0.7° ± 5.4°, *p* = 0.027) (Tables 1 and 2).

### Lower‐limb alignment

In the coronal plane, the FTA showed excellent agreement with the grouping criteria. (186.0° ± 7.5° in varus knees vs. 168.0° ± 9.4° in valgus knees, *p* < 0.001). The MPTA in valgus knees (90.6° ± 6.4°) was higher than in varus knees (81.9° ± 4.4°, *p* < 0.001), while the LDFA in valgus knees (82.7° ± 4.5°) was lower than in varus knees (89.6° ± 3.2°, *p* < 0.001); Furthermore, the aHKA was negative in varus knees (−7.7° ± 5.3°) and positive in valgus knees (7.9° ± 7.0°, *p* < 0.001), indicating distinct coronal plane alignment phenotypes between the two groups. No significant differences were found in sagittal‐plane parameters (knee flexion/extension angle). In the axial plane, tibiofemoral rotation was significantly greater in varus knees (91.8° ± 12.3° vs. 82.5° ± 15.8°, *p* < 0.001), reflecting relative tibial internal rotation in varus knees and external rotation in valgus knees (Tables 1 and 2).

## DISCUSSION

The principal findings of this study are as follows: (1) valgus and varus knees exhibit significantly different morphological characteristics, with valgus knees demonstrating greater femoral neck anteversion and increased distal femoral external rotation, and varus and valgus knees also display a different bowing position of tibia and a different inclination direction of the distal articular surface. (2) Under weight‐bearing conditions, valgus and varus knees exhibited distinct 3D alignment characteristics, including differences in axial tibiofemoral rotation and opposite patterns of distal tibial articular surface inclination, reflecting variations in the lower‐limb functional chain.

The strength of this research lies in its use of CT modelling combined with standing X‐ray fusion, enabling evaluation of lower‐limb alignment and skeletal morphological parameters under functional weight‐bearing conditions. This approach further highlights 3D spatial coupling within the lower‐limb functional chain and the corresponding compensatory mechanisms. Such an integrated spatial analysis cannot be achieved using traditional 2D imaging or non–weight‐bearing CT alone.

In this study, although no significant difference was observed between the two groups in terms of overall femoral bowing, significant morphological differences were identified in the proximal and distal femurs. First, femoral neck anteversion was significantly higher in valgus knees, consistent with the findings of Kawahara et al. [9], that varus knees exhibit approximately 5° less anteversion than normal or valgus knees based on 3D CT analysis. From a biomechanical perspective, this may lead to an axial deviation of the lower limb (knee joint tending toward valgus) and alterations in the patellofemoral joint trajectory. The high femoral anteversion angle is considered an anatomical predisposing factor for lateral subluxation of the patella and patellofemoral asymmetry [12]. Therefore, the increased femoral anteversion observed in valgus knees may reflect a proximal femoral rotational characteristic associated with valgus alignment and may potentially affect patellofemoral joint stability. This finding extends previous knowledge, which was limited to non‐weight‐bearing CT measurements, confirming that valgus knees are still characterized by excessive femoral neck anteversion during weight‐bearing conditions. Distal femoral rotational parameters also differed significantly: valgus knees exhibited greater posterior condylar external rotation compared with varus knees. This finding is consistent with MRI‐based studies: compared to varus knees, valgus knees exhibit approximately 0.5–1° of additional distal femoral external rotation [6]. Although the absolute magnitude of this difference is relatively small, the greater distal femoral external rotation observed in valgus knees suggests that potential differences in the morphological development of the lateral femoral condyle may influence distal femoral torsion. In summary, valgus knees exhibit distinct morphological differences from varus knees in terms of femoral anatomy.

Overall tibial morphological parameters showed no significant differences between the two groups in terms of the degree of tibial shaft bowing (including overall bowing, anterior bowing, and lateral bowing. However, the position of maximum bowing was slightly more distal in the valgus knee group. In contrast, bowing in varus knees tended to be concentrated proximally, potentially reflecting the contribution of proximal tibial shape to knee varus. Although this difference did not reach strong statistical significance, the trend aligns with the clinical understanding that varus deformity of the knee often results from proximal tibial varus [20]. Given the relatively small effect size, this finding should be interpreted as descriptive, and its relevance to clinical management remains to be further determined. However, there was no significant difference in tibial torsion between the two groups, suggesting that axial rotation of the tibia is not a primary factor distinguishing varus/valgus knee deformities. A more obvious difference was observed in the inclination of the distal tibial articular surface. Results showed that varus knees exhibited a valgus inclination of the distal tibial articular surface (approximately +0.9°), whereas valgus knees demonstrated a varus inclination (approximately −0.7°). This difference was statistically significant and is consistent with previously reported compensatory relationships between knee deformity and ankle alignment, whereby varus knees are associated with relative ankle valgus and valgus knees with relative ankle varus [25]. Although the magnitude of the inclination difference was modest, it may reflect coordinated adjustment along the lower‐limb functional chain during weight‐bearing. In this context, our findings provide weight‐bearing 3D quantitative data that are consistent with a previously described compensatory pattern.

Varus and valgus knees also demonstrated substantial differences in lower‐limb alignment. The FTA, defined as the angle between the anatomical axes of the femur and tibia in the coronal plane, showed a significantly greater value in varus knees and a lower value in valgus knees. This pattern follows the expected coronal alignment trend defined by mechanical HKA classification and indicates coherence between anatomical axis alignment and mechanical axis–based grouping [18]. Furthermore, the agreement between FTA and HKA supports the internal consistency of our weight‐bearing 3D assessment framework. Compared with FTA, MPTA/LDFA in the coronal plane better identifies the bony origins of deformity. In this study, valgus knees tend toward “femur‐dominant” (lower LDFA), while varus knees tend toward “tibia‐dominant” (lower MPTA), indicating distinct contributions of the femur and tibia to deformity. These findings are consistent with previous radiographic morphotype studies [22]. Meanwhile, the aHKA, as a phenotypic indicator derived from MPTA and LDFA, can be used to further characterize coronal alignment. In recent years, aHKA have increasingly been used to assess stable mechanical axes following arthritis onset and are associated with individualized alignment strategies [21]. In addition, the reproducibility of aHKA measurement has also been validated on both full‐length radiographs and CT‐based planning systems [7]. In this study, the significant intergroup difference in aHKA further supports that varus and valgus knees are not merely opposite directions on a 2D axis, but rather represent distinct coronal alignment phenotypes with different femoral–tibial contribution patterns. These differences may interact with the multi‐planar characteristics observed in our weight‐bearing 3D assessment. In the sagittal plane, no significant differences were observed between the two groups in knee flexion/extension angle, suggesting that varus and valgus knees exhibit no obvious morphological bias toward hyperextension or flexion contracture. In other words, isolated coronal plane deformities did not result in systematic differences in knee extension during standing between the two groups. This may indicate that regardless of knee varus or valgus deformity, the accompanying degree of sagittal plane compensation (such as mild flexion contracture) was similar. Notably, significant differences were observed in axial rotational alignment. The TEA–PT axis angle is significantly greater in the varus knee group than in the valgus knee group, suggesting that the tibia in varus knees exhibits a more internally rotated orientation relative to the femur, whereas the tibia in valgus knees tends toward external rotation relative to the femur. Previous research of kinematics in varus knees and healthy subjects revealed that changes in the rotational plane of varus knees are primarily driven by the thigh, exhibiting greater external rotation compared to healthy individuals [5]. This observation is consistent with the static alignment findings of the current study. In this research, the tibia in varus knees exhibited a more internally rotated orientation relative to the femur; in other words, the femur showed a greater tendency toward external rotation relative to the tibia. The consistency between dynamic and static findings indirectly demonstrates the accuracy of this research's rotational alignment results for valgus knees, as well as the significant differences in lower limb alignment between varus and valgus knees during weight‐bearing. This outcome may result from structural and compensatory changes arising during prolonged pathological adaptation processes [2]. Consequently, it not only reflects differences in the level of alignment between varus and valgus knees but also indicates that coronal plane deformities and axial rotation of the knee joint are interrelated.

This research has several limitations. First, the 3D–2D registration approach used in this research provides alignment and morphological parameters only under static, weight‐bearing conditions. In clinical settings, lower‐limb alignment and load‐bearing patterns may change during dynamic activities such as walking or single‐leg stance. The absence of dynamic functional assessment may limit our understanding of functional alignment. Second, due to limitations in examination methods, we primarily focus on bony parameters, with insufficient consideration given to soft tissue factors such as cartilage degeneration or meniscal pathology. Additionally, the data were obtained from patients with osteoarthritis, whose bone morphology may differ from that of younger or healthy individuals due to degenerative changes. Therefore, caution should be exercised when generalizing these findings to other populations. Third, the study group exhibited a sample size imbalance, with varus knees being the majority. This reflects the actual clinical distribution. Although effect sizes and confidence intervals were reported for interpretability, residual confounding factors resulting from intergroup imbalance cannot be completely excluded. Future studies with larger valgus cohorts and more comprehensive clinical covariates may benefit from matched or prospective designs. Additionally, incorporating different disease stages and broader populations, combined with kinematic analysis, would further validate and deepen our understanding of the 3D morphological differences between varus and valgus knees.

In summary, this study elucidates the differences in femoral and tibial morphology and lower limb alignment between varus and valgus knees from a weight‐bearing 3D evaluation approach. Our findings emphasize that varus and valgus deformities are not only simple 2D shifts in alignment but also involve complex bone structural remodelling and adaptations. Recognizing the distinct roles of the femur and tibia in different deformities, along with accompanying lower limb changes, this anatomical understanding provides a scientific basis and new perspective for exploring the aetiology of osteoarthritis.

## CONCLUSIONS

Through 3D assessment in the standing position, our research identified weight‐bearing 3D skeletal morphology and lower‐limb alignment in osteoarthritic knees. Compared with varus knees, valgus knees demonstrated greater femoral neck anteversion and distal femoral external rotation. Coronal alignment patterns suggest that valgus knees are more femur‐dominant, whereas varus knees are more tibia‐dominant, corresponding to distinct aHKA phenotypes. In addition, valgus knees exhibited a more externally rotated tibial orientation relative to the femur, whereas varus knees showed relative tibial internal rotation; distal tibial articular surface inclination also showed opposite directions between deformity patterns. These findings demonstrate that valgus and varus knees differ in weight‐bearing 3D skeletal morphology and lower‐limb alignment, supporting a multiplanar understanding of knee deformity patterns in osteoarthritic knees.

## AUTHOR CONTRIBUTIONS

Fangzhou Chi, Tomoharu Mochizuki, Keisuke Maeda and Hiroyuki Kawashima conceived the study. Fangzhou Chi, Tomoharu Mochizuki, Keisuke Maeda and Hiroyuki Kawashima designed the study. Fangzhou Chi, Tomoharu Mochizuki and Keisuke Maeda collected and Fangzhou Chi analyzed the data, and Fangzhou Chi and Tomoharu Mochizuki draughted the initial manuscript. All authors gave critical review and advice on the study design and interpretation. All authors contributed to reviewing and revising the manuscript and agreed on the final draft.

## CONFLICT OF INTEREST STATEMENT

The authors declare no conflicts of interest.

## ETHICS STATEMENT

The Institutional Review Board of our University (Niigata University) approved this study (approval No.2020‐0448). All the participants provided informed consent before participating in the survey. All the participants provided informed consent before participating in the survey.

## Data Availability

The data that support the findings of this study are available from the corresponding author upon reasonable request.
